# A Systematic Review: Costing and Financing of Water, Sanitation, and Hygiene (WASH) in Schools

**DOI:** 10.3390/ijerph14040442

**Published:** 2017-04-20

**Authors:** Shannon M. McGinnis, Thomas McKeon, Richa Desai, Akudo Ejelonu, Stanley Laskowski, Heather M. Murphy

**Affiliations:** 1 College of Public Health, Temple University, Philadelphia, PA 19122, USA; shannonmarcail@gmail.com; 2 Department of Earth and Environmental Sciences, University of Pennsylvania, Philadelphia, PA 19104, USA; mckeont@mail.med.upenn.edu (T.M.); dricha@sas.upenn.edu (R.D.); akudoejelonu@yahoo.com (A.E.); stanlaskowski7@gmail.com (S.L.)

**Keywords:** WASH, school, developing countries, costing, financing, cost

## Abstract

Despite the success of recent efforts to increase access to improved water, sanitation, and hygiene (WASH) globally, approximately one-third of schools around the world still lack adequate WASH services. A lack of WASH in schools can lead to the spread of preventable disease and increase school absences, especially among women. Inadequate financing and budgeting has been named as a key barrier for integrating successful and sustainable WASH programs into school settings. For this reason, the purpose of this review is to describe the current knowledge around the costs of WASH components as well as financing models that could be applied to WASH in schools. Results show a lack of information around WASH costing, particularly around software elements as well as a lack of data overall for WASH in school settings as compared to community WASH. This review also identifies several key considerations when designing WASH budgets or selecting financing mechanisms. Findings may be used to advise future WASH in school programs.

## 1. Introduction

It has been estimated that 10% of the total global burden of disease could be prevented by improvements to water supply, sanitation, and hygiene (WASH) [1]. For example, 88% of diarrhea cases worldwide are attributed to inadequate WASH which results in 1.5 million preventable deaths each year, mainly among children [1]. WASH interventions can significantly reduce both the severity and prevalence of diarrhea, infectious diseases, and some vector-borne diseases and decrease child mortality rates around the globe [1]. Improved WASH can also have economic benefits through reducing medical treatment costs, preventing death, and increasing productivity [1]. Despite these advantages, in many countries around the world, a lack of finances, water quality standards, accountability, management, and a low prioritization of WASH all contribute to an inability to effectively build and maintain water and sanitation services [2], resulting in millions of preventable illnesses and deaths each year [2,3]. For this reason, this review seeks to improve knowledge around the costs of implementing WASH programs and to discuss financing strategies that may be used to meet the needs of WASH in schools globally.

As part of its Millennium Development Goals (MDGs), the United Nations (UN) set out to cut the proportion of people without access to safe drinking water and adequate sanitation in half by 2015 [4]. While these efforts have greatly increased the percentage of people around the globe with access to improved water sources and adequate sanitation since 1990, they have been primarily focused around WASH in the household or community, rather than at the institutional level [5]. As a result, important gaps remain in access to WASH, particularly in school settings [5]. For this reason, as a part of the Sustainable Development Goals following the 2015 MDG deadline, the Joint Monitoring Program (JMP), a collaboration between the World Health Organization (WHO) and the United Nations International Children’s Emergency Fund (UNICEF) to monitor access to drinking water and sanitation globally, has proposed to prioritize improving access to WASH at the institutional level including schools and healthcare facilities [5].

As of 2015, approximately one-third (31%) of schools globally do not have access to adequate water supply and even more do not have access to adequate sanitation (44%) [5]. Inadequate WASH is particularly concerning in schools due to the greater potential for disease transmission among children, who are considered to be a vulnerable group [5,6]. A lack of improved WASH may also contribute to school absences [7] which are associated with reduced academic performance, drop-out rates, and delays in academic and social development [8]. School absences have also been used as a proxy for health status among children in developed countries [9]. The relationship between WASH and school absences is particularly important for menstruating girls who require facilities for personal hygiene [5,7,10,11] and for this reason, WASH interventions in schools may also help reduce gender disparities in school performance and attendance [10].

The costs of implementing improved WASH services are less than the health costs associated with waterborne disease [12]. In fact, the WHO estimates that investment in WASH can lead to economic returns of $2 for every dollar spent on water and $5.50 for every dollar spent on sanitation [13]. Nevertheless, inadequate WASH is still an issue in many countries due to a lack of awareness and government policies, insufficient budget allocations, and financial resources [14]. As a result, in 2014, 80% of countries reported their current levels of financing are insufficient to meet their targets for drinking water and sanitation [15]. This is especially an issue in rural areas that receive less than 10% of WASH financing globally [15]. In order to improve budgeting and decision-making, it is important to understand the costs to implement and maintain WASH programs and infrastructure in developing countries [16]. For these reasons, improving knowledge around cost components and potential methods of financing for future WASH programs may help to support the planning and designing of interventions at the school level. The purpose of this systematic review is to understand what costing data is available on WASH in schools globally, identify financing mechanisms that may help to support funding of WASH in schools, and address key considerations or barriers to take into account when designing budgets or financing models for WASH in schools programs.

## 2. Materials and Methods

### 2.1. Research Questions

In an effort to recover all existing costing and financing data relevant to WASH in schools in a developing country context, two research questions were addressed in this review:

1. What are the elements, and associated costs, that could apply to a WASH in schools programs in a developing country context?
2. How can or how is WASH in schools financed in a developing country context?

The first question seeks to identify the various elements that could be included in a WASH in schools programs along with their associated costs. The second question aims to recover any information available on financing methods used in schools to support WASH.

### 2.2. Review Protocol

A review protocol was developed using the “Cochrane Handbook for Systematic Reviews of Interventions” [17]. In December 2015, literature was searched using the PubMed/MEDLINE database. In an effort to capture relevant “grey literature” and reduce publication bias, Google Scholar, Google, and ProQuest Dissertations and Theses electronic databases were also employed. For the searches performed in Google Scholar and Google, the first 100 relevant articles were exported to Zotero reference software [18,19]. Titles, abstracts, and keywords were searched using terms outlined in Table 1. When performing searches, terms were separated by the Boolean terms OR/AND.

To capture all relevant literature, the search for question 1 was not restricted to WASH in schools, as initial rapid literature scans determined that including the term “school” reduced the number of articles recovered significantly. For question 2, search terms were broadened to include financing models from the healthcare sector that may be applied to WASH in schools. These sources were included to get a broader understanding of possible financing mechanisms as literature around financing of WASH in schools is limited. These terms were selected based on initial rapid literature scans that recovered interesting financial models from the healthcare sector. Only studies published in English were included in this review. The oldest sources recovered in the initial rapid literature scans dated back to 1991, therefore a date range of 1990 to 2015 was selected. Literature recovered from the four databases were exported into Zotero reference software, merged, and de-duplicated.

**Figure 1:**
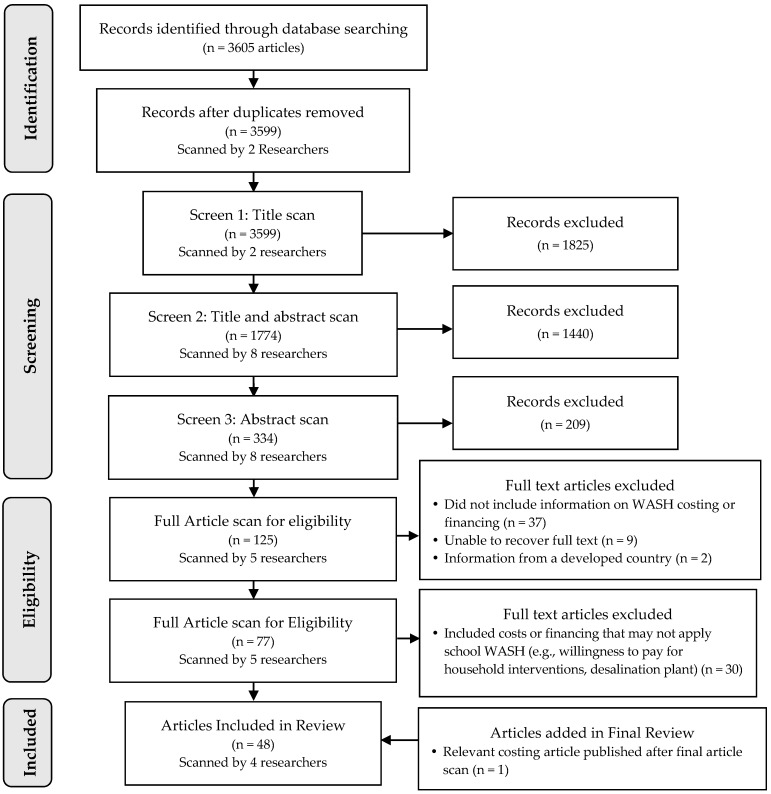
Systematic review article screening process and results for screens 1–5 for the review of water, sanitation, and hygiene (WASH) in schools costing and financing, 1990–2015.

A total of five literature screens were conducted which narrowed down the articles from an initial 3605 to 47. During the screening process, an additional publication relevant to WASH in schools was shared with the research team and included in the final review making the total number of articles screened 48. The screening process and results are illustrated in Figure 1.

In screen 1 and 2, all articles were scanned for titles based on the relevance of inclusion and exclusion criteria specified in Table 2. In screen 3, the inclusion/exclusion process was based on four specific questions outlined below. If the answer to Question 1 (Q1) was a “No”, the article was excluded. If all answers to Q2, Q3, and Q4 were a “No”, then the article was further excluded.

Q1. Is the article set in a developing country context?
Q2. Does the article answer “Yes” to any of the following sub-questions?
  - Does the article outline a specific cost of a WASH component relevant to school WASH?
  - Does the article outline financial aspects of WASH in schools?
  - Does the article outline financial aspects of WASH in a community/village/city?
  - Does the article include a cost-benefit analysis of WASH on public health?
  - Does the article include steps taken to improve WASH in schools?
  - Does the article include steps taken to improve WASH in a community/village/city?
  - Does the article specify elements of WASH in schools?
Q3. Does the article outline programs or financial models for systems in other sectors that may be applied to WASH in schools (e.g., in healthcare)?
Q4. If Y to Question 3,
  - Does the article present specifics costs associated with the program?
  - Does the article propose or describe financing mechanisms?

In scans 4 and 5, the screening data extraction was based on three specific areas: country and setting, WASH elements and costs, and financing mechanisms. Finalist articles were classified into three major categories: (1) Articles that outlined costs of WASH elements relevant to WASH in schools; (2) Articles that outlined either successful or theoretical financing mechanisms relevant for WASH in schools; (3) Articles that discussed both 1 and 2. For each category, there was a set of data extraction questions used to extract relevant information into an Excel database. Data extraction questions involved identifying the specific WASH program elements and their associated costs, breaking down elements into hardware, software, and recurring costs. For financing mechanisms, extraction questions involved identifying whether the mechanisms were successful or theoretical and the types of financing used (i.e., government, private sector, non-governmental organization (NGO) financing, user fees, etc.). Results from the data extraction process were analyzed by category in a qualitative manner highlighting regional trends and identifying data and knowledge gaps in the literature. The remaining sources described either school or community WASH, financing models for WASH, other applicable financing models, or some combination of these. All dollar amounts reported in this review are in USD.

## 3. Results and Discussion

A total of 48 articles retrieved from our search are included in this review. Sources referenced both rural and urban settings and included journal articles, government documents, graduate theses, NGO reports, and books. Among the articles included after the final screen (n = 48), only 12 made some reference to WASH in schools. Of these articles, one focused on hygiene education and latrine cleaning [20], one listed support tools that could be used for WASH in school programs [16], one discussed the benefits of funding school WASH [21], and nine discussed comprehensive WASH (including water supply, sanitation, and hygiene) in schools through case studies in Kenya [22,23,24], the Asian-Pacific region [25], Ethiopia [26], India [27], Uganda [28], Bangladesh [29], and Latin American Countries [30]. These results show a lack of published information around WASH in schools as compared to community WASH.

### 3.1. WASH Costing

**Figure 2:**
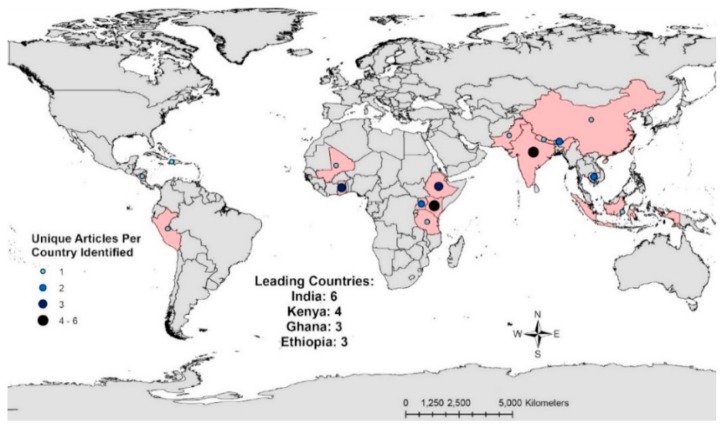
Number of sources with WASH hardware costing data (including recurring costs) per country.

**Figure 3:**
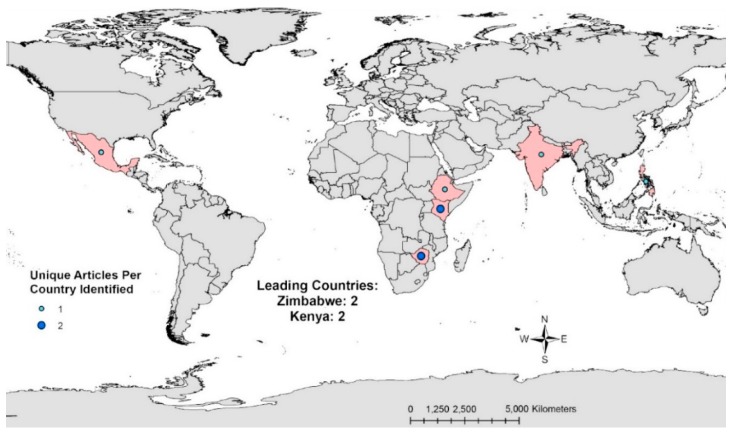
Number of sources with WASH software costing data per country.

In general, there appears to be regional trends in the availability of WASH costing data displayed in Figure 2 and Figure 3. These maps show the number of sources that included WASH hardware costs per country (Figure 2) and WASH software costs per country (Figure 3). Areas where WASH costing data is most concentrated are highlighted in red. The country with the most sources providing hardware costs was India (n = 6), followed by Kenya (n = 4), Ghana (n = 3), and Ethiopia (n = 3) (Figure 2). In total, only seven sources included WASH software costs in six countries. Two of these countries, Zimbabwe and Kenya, had two sources that included WASH software costs while the remaining countries had one source each (Figure 3).

WASH costing data is summarized by category and region in Table 3, Table 4, Table 5, Table 6, Table 7, Table 8, Table 9, Table 10, Table 11 and Table 12. Data in these tables includes the costs provided by the source converted to USD in the “cost” column and the unit provided by the source in the “unit” column. In addition, costs were transformed into a common unit (i.e., per person or per person, per year) and converted into 2015 USD using the World Bank Gross Domestic Product (GDP) deflator. In order to convert volumetric units to costs per person, calculations used use the WHO/UNICEF Joint Monitoring Program’s (JMP’s) definition of “reasonable access” to water as 20 L per person per day [31]. To convert capital costs to costs per person, guidelines from The Sphere Handbook were followed for the maximum number of users per WASH component. These guidelines include 250 people per tap, 500 people per handpump, 400 people per single-user open well, and 20 people per toilet [32]. In order to convert values from a cost per person year to a single cost per person, the lifespan of the technology as prescribed in the sources was used, unless stated otherwise.

For the purpose of this review, WASH costing data was divided into three main categories: hardware costs (i.e., capital costs of infrastructure and hardware), software costs (i.e., education, promotion, administration, and staff costs), and recurring costs (i.e., hardware that needs to be replaced on an ongoing basis, consumables, cleaning, operation, and maintenance costs). Although, some software costs are also recurring, it was decided to group all software costs together to highlight the importance of WASH software components and demonstrate the lack of software costing data recovered. Hardware and recurring data were further grouped into subcategories including: water supply, water treatment and storage, sanitation, and hygiene. Software data were not organized into subcategories due to the lack of available data in this category and the difficulty of organizing program-specific software components.

A total of 22 sources included costs of WASH hardware components, seven sources included costs of software components, and 20 sources included recurring hardware costs. Sources that did not reference a specific country or included data aggregated across multiple regions are included in the “unspecified” country tables (Table 6 and Table 12). In some cases, articles referenced other primary sources for their WASH costing data, indicated in footnotes below.

#### 3.1.1. Hardware

Of all three categories, the most costing information was available for hardware. Hardware costs include one-time costs of building and initiating WASH programs and interventions. The number of sources that included direct costs for WASH hardware totaled 13 for African countries (Table 3), four for Latin American Countries (Table 4), 10 for Asian countries (Table 5), and six for unspecified countries (Table 6). Out of the 101 individual costing data points gathered across all regions, 50 (50%) were for water supply. Hardware costs of water supply included costs for boreholes and tube wells (n = 11), standpipes or communal stand posts (n = 9), rainwater harvesting (n = 8), wells (n = 7), house/private connections to water supply (n = 7), handpumps (n = 4), spring sources (n = 2), small town piped water (n = 1), and Escuela Móvil de Agua y Saneamiento (Mobile School for Water and Sanitation or EMAS) pump (n = 1). Hardware costs for water treatment and storage accounted for 32 (32%) of all cost data gathered. These include filtration (n = 17), water storage jars or bottles (n = 8), disinfection or purification (n = 4), adsorption (n = 2), and an ultraviolet (UV) light source (n = 1). Hardware costs for sanitation accounted for 16 (16%) of all hardware costs. Sanitation infrastructure included costs for latrines or toilets (n = 13) and household sewer connections (n = 3). Finally, hygiene infrastructure accounted for only three (3%) of all hardware costs. Hygiene infrastructure included handwashing infrastructure (n = 2) and a water vessel for menstrual hygiene (n = 1).

#### 3.1.2. Software Costs

WASH software components included hygiene education, health and program promotion, program administration, staffing, and capacity building. Costing data for software was divided into two regions, African countries (Table 7) and all other data (Table 8) due to the scarcity of data. In total, seven sources included data on WASH software costs. Of these, only two included software costs without hardware costs as well, demonstrating the lack of importance typically given to software elements. Of the seven data points available for WASH software costs in Africa, the majority were in Zimbabwe (n = 4) followed by Kenya (n = 2) and Ethiopia (n = 1). Software costs for Africa included hygiene education, health clubs, or health promotion (n = 3), promotion and administration (n = 2), staff costs for a handwashing and safe water system (n = 1), and capacity-building (n = 1). Only three sources included WASH software costs in countries outside of Africa, including data from Mexico, The Philippines, and India (Table 8). These sources included three costing data points for hygiene education (n = 1), promotion and administration (n = 1), and staff (n = 1).

#### 3.1.3. Recurring Costs

Recurring costs include hardware that must be replaced, cleaning costs, consumables, or operation and maintenance costs that must be paid for on an ongoing basis. Some recurring costs will be paid monthly or yearly while others are paid as needed. The number of sources that included recurring costs for WASH totaled 12 for African countries (Table 9), five for Asian Countries (Table 10), four for Latin American Countries (Table 11), and six for unspecified countries (Table 12). Of the 82 recurring cost data points across all regions, 26 (32%) were for water supply. Recurring costs related to water supply included operation or maintenance of water supply (n = 11), cost of water (n = 6), recurrent costs of water supply hardware (n = 5), repairs for hardware (n = 2), energy costs for water supply (n = 1), and a security guard (n = 1). Water treatment and storage made up 29 (35%) of all recurring cost data points. These included disinfection (n = 13), filtration (n = 6), operation and maintenance of disinfection hardware (n = 6), operation and maintenance of filtration hardware (n = 2), cleaning of storage tanks (n = 1), and water testing (n = 1). Costs for sanitation made up 19 (23%) of all recurring cost data. Recurring sanitation costs included cleaning supplies (n = 6), operation and maintenance of latrines or toilets (n = 4), pit emptying (n = 4), latrine maintenance (n = 2), pit latrine additives (n = 1), repairs to latrine door (n = 1), and household sewer connection (n = 1). Costs related to hygiene made up 8 (10%) of all recurring costs data. Recurring data for hygiene interventions was only available for African countries (Table 9). These costs include repairs of handwashing infrastructure (n = 2), toilet tissue (n = 2), soap (n = 2), plastic scoop (n = 1), and sanitary pads (n = 1).

#### 3.1.4. Costing Trends & Gaps

The costs of WASH components varied across regions. For example, the per capita capital cost of a borehole in 2015 USD was $16.96–37.65 in Africa, $69.06 in Latin America, and $21.34–27.90 in Asia (Table 3, Table 4 and Table 5). In addition, the per capita capital cost of a dug well ranged from $26.37–34.33 in Africa, $27.62–36.10 in Asia, and $60.27 in Latin America (Table 3 and Table 4). Table 3, Table 4, Table 5, Table 6, Table 7, Table 8, Table 9, Table 10, Table 11 and Table 12 also showed variations in the types of costing data available by region. For example, while boreholes were the most frequently described water supply source in African countries, house connections, boreholes, and wells were described at similar frequencies in Latin American and Asian countries. Although a lack of available data makes it difficult to compare costs across regions, past studies have supported that costs of WASH elements vary by geographic area due to differing local costs of labor and materials [24,34,46]. Other local factors such as geography, culture, or funding availability may also influence the types WASH components described in each region.

Of all three categories, the most data were available for hardware costs. Within this category, data was more available for water supply (e.g., boreholes, house connections, wells) and water treatment and storage (e.g., filtration, disinfection). The least amount of data was available for hygiene with only three data points available for capital hardware costs and eight data points available for recurring hardware costs for hygiene interventions (e.g., handwashing facilities, water vessel for menstrual hygiene, soap). Evidence suggests that hygiene is particularly important for reducing waterborne disease mortality and morbidity [60], yet these interventions may also be particularly sensitive to cultural norms and educational approaches [16]. Further, only one source included specific costing information around infrastructure designed for menstrual hygiene management [24], which is important for promoting gender equity by reducing school absences among young women [5,7,11]. These results suggest that more costing hardware data is needed around hygiene infrastructure and menstrual hygiene management.

In general, there were large gaps in the availability of software costing data, especially in regions outside of Africa. This finding is consistent with past studies that found implementing software aspects of WASH, such as hygiene promotion, community education, and training programs, were more challenging than implementing hardware aspects, such as installing infrastructure [30]. Unlike hardware costs, most software costing information recovered in this review focused on hygiene programming. In addition, software costs appeared to be specific to individual program designs and therefore may be less generalizable than hardware costs. Despite this, data around the costs of WASH software components is critical for future planning and budgeting for school WASH, as hardware alone is not enough to deliver health benefits of water and sanitation interventions [27].

Including costs for maintaining, repairing, and replacing infrastructure in WASH budgeting is important to ensure that interventions are sustainable in the long-term [46]. Types of recurring hardware costs identified in this review were highly variable, ranging from small cleaning supplies to larger scale repairs, and were dependent on the individual program or intervention strategy. These recurring costs may be difficult to predict during budgeting, which may explain the lack of data on these costs in the literature. Like hardware costs, most recurring cost data was for water supply maintenance and water treatment rather than for hygiene and sanitation. Since ongoing costs of repairs and maintenance may be difficult to account for during planning, it is important for future research to investigate long-term recurring costs of WASH interventions to assist in planning and ensure program sustainability.

### 3.2. Financing

Financial planning is particularly important to ensure WASH facilities are maintained and programs are sustainable [11]. However, many school WASH programs suffer from a lack of financial planning and management [11]. To address this issue, this review gathered information around financing mechanisms and successful financing models that may be applied to WASH in schools. Out of the 48 sources included in this review, a total of 27 included financing models applicable to WASH in schools. Table 13 includes sources that outlined successful or theoretical financing models for WASH in the community or school setting and Table 14 includes sources that outlined potentially applicable non-WASH successful financing models. Successful models are defined as those that have been implemented in the field while theoretical models include aggregated data sets from multiple programs or sources that mention potential financing mechanisms but do not outline specific examples of how these mechanisms have been used previously.

In Table 13 and Table 14, financing mechanisms are divided into three groups: government financing, private or non-governmental organization (NGO) financing, and user fees. Table 13 and Table 14 are organized by whether the source referenced community or school WASH and which combination of the three financing categories was used in the source’s financing model(s). The region with the most sources available for successful or theoretical WASH financing models was Africa (n = 7), followed by Asia (n = 4), and Latin America (n = 1). Ten sources included successful or theoretical WASH financing models for countries in multiple regions or an undefined geographic area. Five sources described financing for WASH in the community setting, three sources described financing for WASH in both communities and schools, and only one source described financing for WASH in schools (Table 13).

Table 14 includes sources that discussed relevant non-WASH successful financing models that may be applicable to WASH in schools. A total of four sources are included in this table; two reference programs in Asian countries and two include information on countries across multiple regions. Types of financing models included were public water supply and water treatment, health care, infrastructure, education, youth development, poverty, social safety net, agriculture, and insurance. Sources that included only challenges or recommendations around financing models and mechanisms were not included in Table 13 and Table 14; however, key findings from these sources are included in the discussion below.

#### 3.2.1. Government Financing

Government financing methods were included in 22 (81%) sources that described successful or theoretical financing models. Types of financing methods included in this category were grants, government budgets, subsidies, taxes, tariffs, constituency development fund (Kenya only), loans from national agencies, public spending, and cost-recovery models. In many cases, government funding is the main financing source for WASH [12]. In addition, in many countries, local governments are also responsible for the delivery of sanitation services and the planning and oversight of some aspects of WASH, such as waste management [21]. However, in most cases the amount of government funds allocated to WASH do not reflect its importance [73].

A lack of political will is a key barrier to encouraging government funding and involvement in WASH [12,21,46]. WASH programs, especially in schools, are often not a priority of local governments due to larger needs [28], competition with other economic sectors [69], limited local resources [28], and public debt [12,74]. Government officials may also feel a lack of accountability and motivation around funding WASH [68]. Further, in many cases, WASH might be divided among many different government sectors [69,77]. While this may allow governments to tap into some important assets (e.g., health sector could provide software through hygiene education and behavior change [77]), dividing WASH among multiple sectors also causes a challenge in promoting a combined and coordinated effort [69,78]. In order to address some of these barriers, it is important to encourage and promote WASH objectives in government planning and budgeting [69]. This can occur through lobbying government officials and relevant donors on issues relating to financing [12] or through advocacy [69]. Because governments are responsible for the management of public resources [28], commitment from local governments around WASH is particularly important for ensuring successful and sustainable programs. According to the most recent UN-Water Global Analysis and Assessment of Sanitation and Drinking-Water (GLAAS) report (2014), roughly one fifth of countries have a plan for WASH in schools that is being fully implemented or funded. Although data suggests that governments have increased spending and allocation of national funds for WASH, gaps remain between country plans and available budgets, with 80% of countries reporting that they have insufficient funds dedicated for WASH [15]. Financing of WASH in schools can be particularly challenging as numerous government ministries may be involved in the delivery of WASH in a school setting. For example, in Mali, four government ministries (Water, Sanitation, Health, and Education) are all key players in the delivery of WASH in schools programs across the country [79].

#### 3.2.2. Private Donor Financing

Private and donor financing methods were included in 18 (67%) financing sources. Financing methods in this category included donors, private sector, NGOs, micro-credit/micro-financing, loans, public-private partnerships, cooperatives, donor grants, and international loans/bonds. Many WASH programs rely heavily on financing from foreign aid and the private sector. For example, at one point donor financing accounted for up to 75% of the funding for the water sector in Uganda [28]. Despite the heavy reliance on this source of financing, aid flow to the developing world has declined over the past two decades [12]. For this reason, international aid is thought to be unable to keep up with additional WASH financing needs due to future population growth [12,50].

In addition to aid, private sector and NGO involvement have been important to the development of many successful WASH programs. This may include international private donors, or domestic private providers such as water vendors [12]. Involving NGOs or the private sector may add support to local projects by bringing in skilled contractors, connecting local areas to companies that provide filtration or disinfection products [68], providing technical support or advice [39], and designing or building infrastructure with long-term benefits [36,39,68] such as drilling wells [39]. Private sector financing might also be useful for relieving governments of a budget deficit [70]. However, using this type of funding for WASH is highly controversial [70]. In some cases, involvement of the private sector has resulted in bribery, corruption, and non-compliance with contractual agreements [70]. In addition, there is often a divide between the goals of private funders and local WASH partners [46,70]. For example, cost recovery is important to private funders [12,69] who are concerned with avoiding financial deficits [68]. However, ensuring cost recovery can be difficult (e.g., water that is consumed without being billed can add to the expenditure without potential for cost recovery [26]). For this reason, the private sector may be more interested in funding the power and gas sectors which have a higher return on investment and more frequent payback periods [12]. Private financers may also be more willing to finance communities with a greater potential for profitability, rather than ensuring access for poor [70] or underserved areas [12], causing most private financing to be concentrated in major cities [21,39]. In addition, private financers may also find other ways to cut costs, such as hiring fewer contractors, which may be detrimental to program efficacy on the local level [46]. As a result many argue that private sector financing may be unable to fulfill the investment needs for WASH on its own [12,21,70].

In order to secure private or donor financing for WASH, it is important to present “implementable programs” with “a focus on cost recovery” [69]. In addition, it is important to ensure that there is transparency and accountability in the way funds are managed and used as well as quality implementation of WASH programs [69]. When international donors or financiers are involved it is also important to ensure that they work at the local level with the government or community leaders to implement locally relevant and sustainable programs [46,68]. Because private and donor financing is often unreliable, it is recommended that projects using on this financing should build plans to be self-financed after the donor or investor leaves to ensure sustainability [46].

#### 3.2.3. User Fees

Finally, 22 (81%) sources included user and household fees as a part of their financing models. This category included user fees, costs for beneficiaries, community financing, credit, school budget, parent-teacher association, fees for parents, service charge, and volumetric based water charges. Keeping financing at the user-level, may help ensure project ownership, making this financing method more likely to be sustainable in the long term [46]. However, this financing mechanism also has some challenges. In most cases, user fees or tariffs are too low to meet the financial requirements of WASH [48,71]. This may be due to a low willingness to pay for these services because beneficiaries might not understand the full benefit of their investment [77] or some households choose to use cheaper or free water sources [36]. Similarly, schools may forego recurring costs, such as costs of water treatment, when they lack finances [57]. There may also be issues with collecting fees [36] and in the school setting it is often difficult to encourage financial support from parents [30]. Finally, securing user fees might be particularly difficult in rural settings where there is a lack of available credit to fill temporary gaps in finances [77]. In order to effectively gather and use user fees, it is recommended to take steps to make sure that fees are not too costly for the poor [21,39] or to provide a menu of different payment options to take into account varying consumer needs [48]. In addition, it is important to take into account users’ willingness to pay for different service types when designing WASH programs [29,40,41,67].

#### 3.2.4. Financing Trends

Due to the limitations of each of the three financing mechanisms described above and the debate around the relative importance of each [76], almost all financing models included in this review used a variety of different financing mechanisms (Table 13 and Table 14). Only one school WASH program used exclusively school fees to fund its initiative, however, this was a private school and this model is likely not generalizable [23]. Examples of successful financing models that used multiple financing mechanisms are described below:

**Community WASH in Ghana**: Government and NGO financing pays for installation costs while the maintenance is to be covered by the community. NGOs often support the communities by paying up to 95% of the borehole cost, while the community raises 5% of the borehole cost [36].
**School WASH in Kenya**: Financing comes from a combination of NGOs who paid for the majority of capital hardware and WASH infrastructure, government offices in the form of the Constituency Development Fund [CDF] (which were in the form of grants so amount differed by school), government resources devolved from the federal to local level, parent teacher associations and school budgets [24].

Many sources directly addressed the importance of using diverse financing mechanisms for WASH [12,27,76,77]. Using multiple financing methods can help to ensure financial sustainability for programs by including back-up mechanisms [68] or financial reserves [76] if one financing stream becomes reduced or unavailable. However, different goals, philosophies, and levels of commitment may limit the ability of the government, private sector and donors, and users to work together [30]. In addition, different funding agencies may have different budgets, rules, and reporting requirements [39]. For this reason, it is important to facilitate communication and coordination between these different groups [80,81] through donor conferences and meetings [26,30], as well as maintain financial transparency [29]. It is also important to consider the local needs and the availability of local resources when selecting different financing mechanisms [77] and to build financing models in a way where projects can become financially self-contained [46].

### 3.3. Considerations for WASH Budgeting and Financing

Due to the large variety of financing mechanisms and models used for community and school WASH, it is difficult to make clear recommendations around financing. In addition, large discrepancies in available costing data and types of costing data by region; make it clear that decisions around budgeting and implementation will largely vary by geographic area, local context, and project needs. Despite this, this review identified several key considerations for WASH budgeting and financing that are important to address to design successful and effective WASH programs.

#### 3.3.1. Addressing Inequities

A lack of equitable coverage for WASH has been noted as a challenge in improving WASH access in many countries [69,71,74]. Both social and financial inequities may decrease access and use of WASH programs and infrastructure and influence hygiene practices [27]. Specifically, poor populations [21,74] and those with disabilities are often ignored in program design [12,27,29]. For example, a study of school WASH in Bangladesh found that there were 135 disabled students in 65 out of the 117 schools included in the study; most of these students used the same toilets as other children and in six of the 65 schools, disabled children did not use the latrine facilities at all [29]. In addition, minorities, and disparaged groups, such as women or those who are HIV positive, are also important to consider when designing WASH and designating funding [27], as sanitation is seen to have a central role in removing gender biases and addressing social equity [21]. Access to WASH is also limited in rural areas [27,74]. It is estimated that 80% of people without access to sanitation live in rural areas and one-third of rural residents lack access to improved drinking water sources [82]. This is particularly an issue in areas where rural populations are growing, creating an expected increase in future demand [73].

Despite the need to improve WASH access to disparaged or underserved groups, our search found no costing data for targeting underserved populations or building infrastructure that can be accessed by those with disabilities. However, sources did provide some suggestions for allowing for more equitable access. These suggestions include prioritizing underserved populations [12] and reallocating investments to reach these groups [12,27,80]. To ensure access for poor populations, WASH programs may be integrated into poverty reduction strategies or other national planning initiatives [12,21,28]. For example, the poverty eradication plan, or (PEAP) in Uganda, gives priority to water supply and sanitation as an intervention area for poverty eradication [28]. Encouraging poor communities to participate in WASH planning and decision making [12] and providing subsidies for the poor to access services [41] may also increase equity in WASH programs. Further, it is important for future WASH programs to consider using alternative technologies for students with disabilities that would allow them to also access WASH infrastructure [29].

#### 3.3.2. Community Involvement and Education

Several sources identified the need for more community involvement in WASH planning and implementation to ensure more effective [28,50] and sustainable programs [30]. In fact, almost every source included in the review named sustainability of WASH to be a pressing challenge that is important for ensuring that the WASH infrastructure does not “fall into disrepair” [30]. Involving the community was suggested to be one of the most important methods to improve sustainability by making users more responsible for the operation and maintenance of programs once donors or private financers are no longer involved [28]. By encouraging community involvement, it may be easier to take advantage of local resources, build local capacity for WASH, ensure user satisfaction [38], and involve underrepresented groups such as women [49]. For this reason, decentralized decision making for WASH is supported by many NGOs who support ownership and management of projects to the lowest possible level [46]. Strategies to facilitate community involvement may include community mobilization through health clubs, community groups, meetings [26,27] or school WASH committees [29]. Only one source in our review included costing data around community health clubs in Zimbabwe which ranged to approximately $0.21–0.67 per beneficiary or up to $1.40 when including the cost of staff [60].

Raising awareness is also an effective method for creating demand for better sanitation [73] and is known to promote sustainability [81]. Effective strategies to educate community members about WASH can both encourage community involvement and facilitate behavior change [26,27]. In fact, some argue that educational programs that encourage behavior change are necessary to ensuring success of WASH programs and are the most effective method to improve hygiene, especially among women [46]. Two sources are referenced in costing data for hygiene education programs. One, in Mexico, was estimated to cost approximately $2–5 per household per year which included the cost of carrying out ongoing campaigns and maintaining contact with target clients [64]. The other source, in Mekelle City, Ethiopia, cost approximately $31,000 for a three-day educational event for all current primary and secondary school teachers in the city [26]. These very different cost estimates and education methods further highlight the importance of local context in program planning.

In order to both educate and involve the local community in WASH, it is recommended to use a mix of different media types [27,28] and to use lessons from commercial advertising to reach a wide range of audiences [27]. Tools such as social marketing and communication for behavior change are key aspects of successful WASH programs [46]. Advocacy is another recommended strategy that may encourage community involvement [69]. In school settings, encouraging education, leadership, and advocacy among school children is suggested to encourage youth involvement [27]. In addition, integrating WASH into the school curriculum may also help encourage and educate students on the purpose and proper use of new infrastructure or programs [28]. It is also suggested to inform school management committees about new WASH programs [24,29], and to involve both parents and students to facilitate effective community involvement around school WASH [29].

#### 3.3.3. Monitoring and Evaluation

The integration of monitoring and evaluation into WASH programs is recommended to improve efficiency and quality of WASH services [12,16,28,50]. A lack of monitoring and evaluation may lead to poor construction or dysfunctional infrastructure (e.g., due to full pit or septic tanks, chocked pan/pipes, wrong location, etc.) [27]. Monitoring and evaluation may also help to reduce environmental impact [12], foster more efficient spending and budgeting [21], and ensure financial viability and sustainability [68]. Because of this, several sources identified a need for improved monitoring and evaluation [12,21,27,38] as well as for improved evaluation metrics [29]. This review found no data on the costs of monitoring and evaluating WASH programs. Despite this, two sources included examples of monitoring strategies such as household surveys [21], or in the case of school WASH, unannounced school visits [30]. Other methods such as using a state mandated social audit and encouraging community level monitoring have also been suggested [27].

Barriers to monitoring and evaluating WASH programs may include a lack of capacity and knowledge around these strategies [27]. To address this issue, it is important to develop useful and relevant indicators of success and integrate evaluation into the training of WASH staff or managers [30]. Further, it is recommended for programs to keep track of both process and outcome measures and to include incentives for positive outcomes [46]. Finally, developing common metrics, used to evaluate WASH programs that address behavior change, long-term adoption, and sustained use may improve monitoring and evaluation across different projects and settings [38].

#### 3.3.4. Management

Related to monitoring, sources also identified a need for better management when designing WASH programs [24,26,29,38,74]. This can include the operational management of programs and infrastructure [24,26,29,38] as well as financial management [26]. Responsibility for managing WASH programs and infrastructure may fall on the national or local government or the local community [28]. Improving management of WASH programs and infrastructure can enhance efficiency [26,74], control waste, and ensure more timely repairs [26]. In addition, including management in the budgeting and financing scheme can save future costs. For example, in Trinidad, water shortages that occurred due to leakages were attributed to a lack of routine maintenance by engineers and politicians [67]. Eleven sources in our review included costing data on operation and maintenance of WASH infrastructure including repairs, pit emptying, and the cost of a security guard, reflecting the importance of proper management of programs and infrastructure. Despite this, there remains a need for increased budget allocations and attention towards program operation and management [67,74].

Effective financial management can improve accountability, transparency, and cost-recovery [39]. For example, more open financial reporting will ensure that payments go towards maintenance for improving WASH [73]. Better management of user fees can result in more efficient revenue collection and billing for services [26,36]. Financial management assistance at the sector level can also help to avoid fragmented projects without lasting service improvements [69]. Finally, improved financial management may also help WASH programs to pay creditors and avoid losing credibility with the banking community which may create difficulties in receiving future financing [67]. However, despite these benefits, no sources in this review provided costing information for financial management of WASH.

In order to ensure there is sufficient financial support for WASH management it is important to include the costs of operation and management of programs and infrastructure in budget allocations [24,74]. In addition, it is important to build capacity for effective program [69] and financial management in program design [68,73]. To improve community involvement in WASH management, programs may choose to create a system to pay community members for maintenance services [36] or provide financial incentives to grassroots workers [27]. For financial management, it is important to improve institutional framework in budgetary management [74] and to consider involving the local government to assist in financial audits and provide management and technical advice [73].

#### 3.3.5. Lack of Guidance and Technical Assistance

Inadequate data and information [28], a lack of technical support [80], and a lack of trained staff [68] may be important barriers when implementing WASH programs and infrastructure. For example, there is a lack of information around the cost-effectiveness of different intervention strategies which is important for budgeting and decision making [38]. Further, it is often difficult to gather information on school budgeting for WASH, as one source noted relying on the head-teacher or principal accounts and could not get specific data to guide the programs [24]. Overall, this review found a lack of costing data available for school WASH, highlighting a need for better guidance, information sharing, and technical assistance.

To improve the available data and technical assistance to guide future WASH programs, it is recommended for current programs to share and document best practices [38,68] including WASH programs that have been evaluated [68]. This can be accomplished through better information sharing that is available both online and in hard copy for areas without internet access [16] and including tools for implementation, formative research, information on selecting infrastructure, etc. [38]. In addition, local training programs may be offered to improve financial management and program leadership [30] and to help key players such as technicians, health professionals, and social development specialists to work together [21]. For example, the government of India includes mandatory training of central and state officials engaged in the sanitation sector and ensures funding is available for those trainings [27]. Other successful WASH models have also integrated technical assistance for implementers [46]. Similarly, one source suggested improving human resources and encouraged hiring individuals with backgrounds in community development or engineering [68].

## 4. Conclusions

The goal of this research was to conduct a systematic review of the literature to understand what costing data is available and what financing mechanisms have been used or could be used to help support WASH programs in schools. Our search found a lack of WASH costing data overall and identified large gaps in the available costing data for hygiene infrastructure (handwashing with soap, menstrual hygiene management) and software programming (hygiene education, monitoring and evaluation, training, curriculum development, project management). Further, the majority of available costing data for WASH was centered around the African region, demonstrating a need for more information in other geographic areas.

The costing data recovered in this review may be helpful for high-level planning and costing of WASH in schools. These data may assist government officials and others involved in education sector planning to conceptualize the various components of WASH programs that are needed for their local programs and subsequently estimate costs and allocate sufficient funds to meet these needs. The authors acknowledge that the data recovered in the review is limited and there remain significant regional gaps in costing data for certain hardware and recurring costs as well significant gaps in costing of software activities such as: hygiene education, program coordination/management, and menstrual hygiene management. This is consistent with previous research that reports a lack of useful costing information available to make decisions around WASH [16]. Because costs will likely vary by country and within country, more local data is ideal for any planning activities. Nevertheless, the information presented herein may be a good starting point for planning and advocacy purposes. This research highlights the need for costing information to be collected, documented, and published in accessible forums so that those in the WASH and education sectors may use the information in planning of school WASH programs.

In addition, our review recovered a variety of successful and theoretical financing models that have been used to fund community and school WASH programs or other relevant non-WASH programs. The three main financing mechanisms identified were: government and public financing, private and donor financing, and user fees. Due to the benefits and drawbacks of these three methods, almost all sources used a variety of different mechanisms for their financing models. Further, because financing decisions depend largely on program goals, local context, and available resources, it is difficult to identify specific recommendations for WASH financing globally.

Finally, we identified key considerations when designing budgets and selecting financing mechanisms for WASH in schools. These considerations included: addressing inequalities, involving and educating the local community, including effective monitoring and evaluation, integrating strategies for proper program and financial management, and improving information, guidance, and technical assistance for new programs and managers.

Although the current WASH literature provides some costing data around WASH hardware and software components, more information is needed to guide future WASH programs in the school setting. Future evaluations of WASH intervention strategies in schools should also include detailed documentation of program costs, as well as an examination of the financing schemes used to ensure program sustainability. Lessons learned from WASH implementations need to be documented to improve data sharing and decision-making around WASH programs globally.

## Figures and Tables

**Table 1:** Search terms used in systematic review of literature on water, sanitation, and hygiene (WASH) in schools costing and financing, 1990–2015.

**Question 1**	**WASH Terms**	**Cost/Financing Terms**	**WASH Element Terms**
What are the elements, and associated costs, that could apply to a WASH in Schools program in a developing country context?	water; sanitation; hygiene	tariff; price; scale; scaling; “cost structure”; investment; money; cost; financing; finance; “cost effective”; costing; microfinance; subsidies; subsidy; loan; loans; capital; recurring	“operation and maintenance”; training; evaluation; replacement; “water supply”; sanitation; hygiene; latrine; “point of use”; “household water treatment”; borehole; soap; “rainwater harvesting”; chlorine; hardware; software; education; “teacher training”; “menstrual hygiene management”; “cleaning materials”; “cleaning staff”; “hand washing”; construction
**Question 2**	**WASH Terms**	**Cost/Financing Terms**	**Community/School Terms**
How can or how is WASH in schools financed in a developing country context?	water; sanitation; hygiene	“willingness to pay”; finance; “microfinance”; ”public sector finance”; “private sector finance”; “financial model”; government finance; financing; subsidies; “official development assistance” scaling; taxes; tax; school fees; tariff	community; schools; school; local; decentralized; centralized, “parent teacher association”; clinic; hospital; healthcare

**Table 2:** Inclusion and exclusion criteria used during systematic review of literature for screens 1–4.

Research Question	Population	Cost/Financing	Schools	Include
Question 1	Must be representative of a developing country context	Identifies specific costs of WASH program elements	Must be relevant to schools	If it specifies costs or elements of WASH in schools such as training, hardware, software, monitoring, admin costs, evaluation costs, etc.
Question 2	Restricted to following regions: Africa, Asia, South/Central America	Identifies sources of funding/financing for WASH or another sector that could be applied to WASH	Financing mechanisms need to be relevant for schools (i.e., personal microfinance excluded)	If it mentions source of financing in schools, evaluates school WASH, evaluates financing of a health care program or system or community program that could potentially be applied to school WASH

**Table 3:** WASH Hardware Costs in African Countries.

	Hardware Type	Cost	Unit	Per Person 2015 USD	Country	Source
Water Supply	**Boreholes/Borewells**	$1.70	per person, per year	$37.65	Multiple	[33]
	For school WASH	$2.38	per student, per year ^1^	$24.05	Kenya	[24]
		$23	per person	$28.88	Multiple ^2^	[34]
	Fitted w/handpump	$42	per person	$27.49	Ghana ^3^	[35]
		$7605.93	per borehole	$16.96	Ghana ^2^	[36]
		$8316	per borehole	$19.79	Ethiopia ^4^	[26]
	Tube well	$15–40	per person ^5^	$16.73–44.60	Uganda	[34]
	Tube well	>$150	per person ^5^	$167.27	Ethiopia	[34]
	**Public Standpipe** Communal stand post	$2.40	per person, per year	$50.35	Multiple	[33]
	Standpipe	$31	per person	$38.92	Unspecified	[34]
	Service connection-Standpipe	$282	per connection	$80.23 ^6^	Ethiopia ^7^	[26]
	Town water	$70–90	per person	$78.06–100.36	Ethiopia	[34]
	**Handpump** EMAS pump (PVC piston pump) ^8^	$43 or less	per pump	$0.09	Uganda	[37]
	Rope pump (low cost handpump)	$80–100	per pump	$0.16–0.21	Uganda	[37]
	Community handpump	$150–1000	for 250 people or more	$0.62–4.11	Uganda	[37]
	**House Connection**	$102	per person	$128.07	Unspecified	[34]
		$111	per household	$30.94	Ethiopia	[26]
	**Rainwater Harvesting**	$30	per person	$33.45	Uganda	[34]
		$49	per person	$61.52	Unspecified	[34]
	Gutters for rainwater catchment	$0.05	per student ^1^	$0.51	Kenya	[24]
	Water tank for rainwater catchment	$2690	per year, 10,000 L tank ^1^	$0.68	Kenya	[24]
	Water tank for rainwater catchment	$6726	per year, 25,000 L tank ^1^	$1.70	Kenya	[24]
	**Small town piped water**	$79	per person	$86.18	Ghana	[35]
	**Spring source with gravity distribution**	$15	per person	$16.73	Uganda	[34]
		$17–20	per person	$18.96–22.30	Ethiopia	[34]
	**Wells**					
	Dug well	$1.55	per person, per year	$34.33	Unspecified	[33]
	Dug well	$21	per person	$26.37	Unspecified	[34]
	Protected well	$2401.87	per protected well	$8.93	Ghana	[36]
Water Treatment and Storage	**Filtration**					
	Biosand filter	$13	per filter	$3.78	Kenya	[38]
	Biosand filter	$15	per filter with 50 L plastic bucket	$4.36	Ghana	[38]
	Ceramic pot filter	$15–20	per filter	$4.36–5.82	Ghana	[38]
	Ceramic candle filter	$13	per unit, $2 per candle	$3.78	Kenya	[38]
	Ultra-Filtration system	$4003.12	per filtration system, 20 m^3^/day capacity	$4.46	Ghana	[36]
	**Disinfection**					
	Household chlorination	$7	per person	$2.04	Kenya	[38]
	**Water storage jar**					
	Clay jar	$5	per 40 L	$2.74	Ethiopia ^9^	[39]
	Clay pot	$6.80	per 40 L ^10^	$4.12	Kenya	[23]
	Drinking water vessels	$90	unit cost ^1^	$0.07	Kenya	[24]
	Plastic water bottle	$0.19	per 500 mL	$7.68	Kenya	[20]
	Plastic tank	$16	per 100–220 L	$1.76–3.88	Kenya	[23]
	Safe storage-modified clay pot	$6	per pot	$1.75	Kenya	[38]
	Safe storage-modified clay pot with ½ inch brass tip or plastic safe storage container with ½ brass top	$8.50	per container	$2.47	Ghana	[38]
Sanitation	**House connection to simplified sewer system**	$1263	per household	$367.45	Ethiopia	[26]
	**Latrine**s					
	Communal toilet with septic tank	$1053	per toilet with septic tank	$61.27	Ethiopia	[26]
	Household latrines	$13	per latrine	$3.45	Mali	[40]
	Pit latrines	$42	per latrine	$2.44	Ethiopia	[26]
	Pit latrines	$272	per latrine	$14.93	Tanzania ^11^	[41]
	Pour-flush latrines	$789	per toilet with septic tank	$45.91	Ethiopia	[26]
	VIP latrines (4 cabins)	$8965	per unit in a school ^1^	$2.26	Kenya	[24]
	**Handwashing**					
Hygiene	Handwashing station	$57	per station	$0.36	Kenya	[23]
	Handwashing vessels	$224	per unit ^1^	$0.19	Kenya	[24]
	**Water vessel for menstrual hygiene**	$12	per unit ^1^	$0.01	Kenya	[24]

^1^ Source divided capital costs by ten years; ^2^ Citing: WHO/UNICEF, 2000 [31]; ^3^ Source estimates one well or borehole per 300 people, per the government of Ghana, converted to 500 people per borehole per source above; ^4^ Citing: Ministry of Finance and Economic Development and UNDP (2004), [42]; ^5^ For approximately 500 households; ^6^ Per connection, cost per person determined if price per connection was to a household with 4 people per household. Connections may also be to schools, hospitals, etc.; ^7^ Citing: Africa Infrastructure Country Diagnostic [AICD], 2008 [43]; ^8^ EMAS = Escuela Móvil de Agua y Saneamiento or Mobile School for Water and Sanitation, PVC = polyvinyl chloride; ^9^ Citing: Boelee, 2008 [44]; ^10^ Source cost is $34 for 5 pots; ^11^ Citing: Jenkins, Cumming, & Cairncross, 2014 [45].

**Table 4:** WASH hardware costs in Latin American countries (including Mexico, South America).

	Hardware Type	Cost	Unit	Per Person 2015 USD	Country	Source
Water Supply	**Boreholes/Borewells**	$55	per person	$69.06	Unspecified	[34]
	**Handpump** EMAS pump (PVC piston pump) ^1^	$30–45	per pump	$0.06–0.09	Bolivia ^2^	[37]
	**House connection**	$144	per person	$180.80	Unspecified	[34]
	**Public standpipe**	$41	per person	$51.48	Unspecified	[34]
	**Rainwater harvesting**	$36	per person	$45.20	Unspecified	[34]
	**Wells**					
	Dug well	$48	dug well, per person	$60.27	Unspecified	[34]
Water Treatment and Storage	**Filtration**					
	Ceramic candle filter system	$21	per system	$6.11	Bolivia	[38]
	Ceramic pot filter	$7	per filter	$2.04	Nicaragua	[38]
	Purification Initiative	$5600	400 purifiers ^3^, training community members and health technicians, and annual salaries for two technicians	$4.76	Haiti	[46]

^1^ EMAS = Escuela Móvil de Agua y Saneamiento or Mobile School for Water and Sanitation, PVC = polyvinyl chloride; ^2^ Citing: MacCarthy, Buckingham, & Mihelcic, 2013 [47]; ^3^ Purifier is a two-bucket system that includes a polypropylene string-wound filter in the top bucket and a granulated activated-carbon filter in the bottom bucket. Chlorine is added by users.

**Table 5:** WASH hardware costs in Asian countries.

	Hardware Type	Cost	Unit	Per Person 2015 USD	Country	Source
Water Supply	**Boreholes/Borewells**	$1.26	per person, per year	$27.90	Multiple	[33]
		$17	per person	$21.34	Unspecified	[34]
	**House connection**	$92	per person	$115.51	Unspecified	[34]
		$38.25	per household	$12.71	India ^1^	[48]
	**Public standpipe**	$64	per person	$80.36	Unspecified	[34]
	Communal stand post	$4.95	per person, per year	$103.85	Multiple	[33]
	Rainwater harvesting	$34	per person	$42.69	Unspecified	[34]
	**Wells**					
	Dug well	$1.63	per person, per year	$36.10	Multiple	[33]
	Dug well	$22	per person	$27.62	Unspecified	[34]
Water Treatment and Storage	**Adsorption**					
	Adsorption column ^2^	$700	per well	$1.51	India	[49]
	Adsorbent media materials	$570	per well-head arsenic removal unit	$1.23	India	[49]
	**Filtration**					
	Biosand filter system	$15–20	for biosand filters, ceramic candles, carbon filtration and resin adsorption units	$4.36–5.82	China	[38]
	Biosand filter	$67	per household	$19.93	Cambodia	[46]
	Ceramic pot filter system	<$10	for ceramic pot filters, ceramic candle filters, silver impregnated foam or ceramic balls and low-end UV	$2.91	China	[38]
	Hybrid ceramic candle filtration and carbon filtration	$15.50	per unit	$4.51	China	[38]
	Hybrid UV with ceramic and carbon filtration units	>$100	per unit	$29.09	Unspecified	[38]
	Filtration plus ozonation	>$100	per unit	$29.09	Unspecified	[38]
	Reverse osmosis	$100–300	per unit	$29.02–87.28	Unspecified	[38]
	**Disinfection**					
	Chlorination	$0.20	per 1 Piyush bottle ^3^	$0.01	Nepal	[25]
	Iodine based disinfection	$32–103	per unit	$9.31–29.97	Unspecified	[38]
	**Water Storage Bottles**					
	Plastic bottles for solar disinfection	$0.80	per person	$0.93	Indonesia	[46]
Sanitation	**Household Sewer Connection**	$68.85	per household	$22.88	India ^1^	[48]
		$100	per household ^4^	$34.82	Pakistan ^5^	[50]
	**Toilets**	$10	per toilet	$0.56	India	[25]
		$10–$1000	per private toilet	$0.61–60.57	India	[51]
	Ecosan Toilet	$96	per household	$27.93	Rural India ^6^	[52]

^1^ Citing: Altaf, 1994 [53]; ^2^ Stainless steel (SS-304) adsorption column with valves, internals, water meter and connections with the existing well-head hand pump, for arsenic removal; ^3^ 0.5% chlorine solution, 60 milliliter dropper bottles, 3 drops per L; ^4^ Reduced cost from standard $1,000 per household; ^5^ Citing: Hasan, 1990 [54]; ^6^ Citing: McCann, 2005 [55].

**Table 6:** WASH hardware costs in unspecified countries.

	Hardware Type	Cost	Unit	Per Person 2015 USD	Country	Source
Water Supply	**Handpump**					
	On drilled well	$17–55	per person	$21.34–69.06	Rural Areas ^1^	[56]
	**Public Standpipe**	$101,149.74–359,070.38	per village ^2^	$30.64–76.59	Unspecified	[48]
	Standpost	$31–64	per person	$38.92–80.36	Rural Areas ^1^	[56]
	**House connection**	$484,608–1,051,793	per village ^3^	$318.56–366.94	Unspecified	[48]
		$92–144	per person	$115.51–180.80	Unspecified ^1^	[56]
	**Rainwater harvesting**	$34–49	per person	$42.69–61.52	Rural Areas ^1^	[56]
	**Wells**					
	Dug well	$21–48	per person	$26.37–60.27	Rural Areas ^1^	[56]
Water Treatment and Storage	**Filtration**					
	Biosand filter	$25–100	per filter ^4^	$6.86–27.44	Multiple	[57]
	Ceramic filter	$8–10	per filter unit	$2.20–2.74	Multiple	[57]
	Ceramic candle filters	$5–10	per filter	$1.45–2.91	Multiple	[38]
	**UV light source**	$10–200	per unit with transformer and electric cord	$2.91–58.19	Unspecified	[38]
Sanitation	**Latrine**					
	Pour-flush latrine	$50–91	per household	$15.69–28.56	Multiple ^1^	[52]
	Pour-flush latrine	<$100	per unit	$6.72	Unspecified ^5^	[50]
	Simple pit latrine	$26–60	per household	$8.16–18.83	Multiple ^1^	[52]
	VIP latrine	$50–57	per household	$15.69–17.89	Multiple ^1^	[52]

^1^ Citing: World Health Organization/United Nations International Children’s Emergency Fund (WHO/UNICEF), 2000 [31]; ^2^ $101,149.74 for a small village, 252,424.42 for a medium village, 359,070.38 for a large village; ^3^ $484,608 for a small village, $715,737 for a medium village, $1,051,793 for a large village; ^4^ Or 0.001–01 $/L; ^5^ Citing: Kalbermatten, et al., 1982 [58].

**Table 7:** WASH software costs in African countries.

Software Type	Cost	Unit	Per Person 2015 USD	Country	Source
**Hygiene education**	$31,000	for a 3 days education program for all primary and secondary school teachers in Mekelle City, Ethiopia	$16.77 ^1^	Ethiopia	[26]
**Health club/Health promotion**	$0.21	average cost per beneficiary	$0.26	Zimbabwe	[59]
**Health club**	$0.67	average cost per member ($1.40 when including staff salaries)	$0.81	Zimbabwe ^2^	[60]
**Promotion and administration** (government-run rural sanitation program)	$16.80	per latrine	$1.14	Zimbabwe ^3^	[60]
**Promotion** (of latrines via health clubs, health staff)	$2.24	per household member (or $13.43 per latrine)	$2.71	Zimbabwe ^1^	[60]
**Staff**	$143	per month, to monitor handwashing and safe water system	$1.37	Kenya	[23]
**Capacity-building**	$334	per year ^4^	$0.84	Kenya	[24]

^1^ Calculated using number of teachers in Mekelle City, Ethiopia reported in the 2009 Education Needs Assessment Report (2016 teachers) [61]; ^2^ Citing: Waterkeyn, 2003 [62]; ^3^ Citing: Cairncross, 1992 [63]; ^4^ Capacity building on the use of infrastructure and WASH monitoring for teachers, PTAs, and government officials, as well as capital software and direct support.

**Table 8:** WASH software costs in other countries.

Software Type	Cost	Unit	Per Person 2015 USD	Country	Source
Hygiene education	$2–5	per household per year ^1^	$1.63	Mexico	[64]
Promotion and administration (government-run rural sanitation program)	$20	per latrine	$1.36	The Philippines ^2^	[60]
Staff	$37.57	per month for 2 persons	$0.16	India	[25]

^1^ Includes costs of carrying out ongoing campaigns and maintaining contact with target clients; ^2^ Citing: Cairncross, 1992 [63].

**Table 9:** WASH recurring costs in African countries.

	Recurring Cost Type	Cost	Unit	Per Person, per Year 2015 USD	Country	Source
	**Cost of water**					
Water Supply	House connection	$0.30	per m^3^	$2.75	Unspecified ^1^	[60]
	House connection	$3.99–5.32	per household, per month	$14.50–19.34	Nigeria	[48]
	Public tap	$1.90–2.30	per month, per household	$6.91–8.36	Nigeria	[48]
	Water truck	$5.68–6.85	per m^3^	$46.22–55.79	Ghana	[36]
	**Energy cost for small town water supply**	$0.21–0.90	per m^3^	$1.67–7.17	Ghana	[65]
	**Operation of water supply**					
	Borehole and handpump (plus “minor” maintenance)	$0–2	per person, per year	$2.18	Ghana	[35]
	House connection	$10.95	per person, per year ^2^	$12.46	Unspecified	[60]
	Maintenance of water source	$56	per school, per year	$0.14	Kenya	[24]
	Rural water system	$2527	per month, capital and operating costs	$7.35	Nigeria	[48]
	Small town water supply	$0.61–2.30	per m^3^	$4.86–18.32	Ghana	[65]
	Small town piped supply	$2.30	per person, per year	$2.51	Ghana	[35]
	**Repairs of hardware**					
	Tap, pipes, gutter repair	$45	per school, per year ^3^	$0.11	Kenya	[24]
	Water hardware	$45	per school, per year ^4^	$0.11	Kenya	[24]
	**Security guard**	$202	per school, per year	$0.51	Kenya	[24]
Water Treatment and Storage	**Cleaning of storage tanks**	$45	per school, per year	$0.11	Kenya	[24]
	**Disinfection**					
	Solar disinfection	$0.16	per bottle ^5^	$1.87	Kenya	[38]
	Water treatment solution (Waterguard)	$0.57	per bottle ^6^	$0.31	Kenya	[23]
	Disinfectant and detergent	$121.00	per school, per year	$0.30	Kenya	[24]
	Water treatment	$40.00	per school, per year	$0.10	Kenya	[24]
	**Operation and maintenance- disinfection**					
	Household chlorination	$3.60	per family, per year ^7^	$1.05	Kenya	[38]
	Solar disinfection	$6.40	per family, per year ^8^	$1.86	Kenya	[38]
	Coagulation/flocculation	$14.60	per family, per year ^9^	$4.25	Kenya	[38]
	Coagulation/flocculation plus chlorine disinfection	$73	per family, per year ^10^	$21.24	Kenya	[38]
	Filtration, disinfection, aesthetics, storage	$3.60	per family, per year	$1.05	Kenya	[38]
	Ceramic candle filter	$1.90	per candle	$1.11	Kenya	[38]
	**Operation and maintenance-filtration**					
	Biosand filter	$0.12	per family, per year	$0.03	Kenya	[38]
	Ceramic candle filter	$4.00	per family, per year	$1.16	Kenya	[38]
Sanitation	**Cleaning**					
	Bleach	$2.80	per bottle	$0.04	Kenya	[20]
	Brooms	$1.75	per broom	$0.03	Kenya	[20]
	Brooms	$13.50–30	per school, per year	$0.03-0.08	Kenya	[24]
	Buckets	$1.40	per 10 gallon bucket ^11^	$0.04	Kenya	[20]
	Buckets and brooms	$20	per school, per year	$0.05	Kenya	[24]
	Hand brushes	$0.50	per brush	$0.01	Kenya	[20]
	**Latrine maintenance**	$1.54	average spent on household latrinerepairs ^12^	$0.41	Mali	[40]
	Latrine repairs	$60	per school, per year	$0.15	Kenya	[24]
	**Operation, Maintenance, Administration**					
	School latrines	$16,800	per year excluding cleaning ^13^	$2.20	Ethiopia	[26]
	Repairs for sanitation hardware	$54	per school, per year	$0.14	Kenya	[24]
	**Repairs to latrine door**	$73–80	per school, per year	$0.18–0.20	Kenya	[24]
	**Pit emptying**					
	Manually with a bucket	$1.35	per year, per user	$1.48	Tanzania	[41]
	Through diversion	$0.68	per year, per user	$0.75	Tanzania	[41]
	Emptying service	$2.20	per month, per property owner	$10.43	Tanzania	[41]
	Pit emptying	$38	per school, per year (one pit)	$0.10	Kenya	[24]
	**Pit latrine additives**	$17	per treatment ^14^	$0.47	Tanzania	[41]
	**Repairs**					
Hygiene	Handwashing taps	$9	per school, per year	$0.02	Kenya	[24]
	Handwashing hardware	$31	per school, per year	$0.08	Kenya	[24]
	**Soap**	$7.30	per 3.5 kg bag Omo powdered soap	$0.11	Kenya	[20]
		$60	per school, per year	$0.15	Kenya	[24]
	**Sanitary pads**	$60	per school, per year	$0.15	Kenya	[24]
	**Plastic scoop (for soap)**	$0.12	per 1 cup scoop	$0.002	Kenya	[20]
	**Toilet tissue**	$29.92	for 150 rolls of toilet tissue per pupil, per term ^15^	$0.30	Kenya	[20]
		$325	per school, per year	$0.82	Kenya	[24]

^1^ Citing WHO/UNICEF, 2000 [31]; ^2^ Assumes a mean daily water consumption of 100 L per capita by those with household connections; ^3^ For rainwater catchment; ^4^ Includes either a borehole or rainwater catchment with gutters and four storage tanks for schools where a borehole could not be built; ^5^ Assumes 40 2-L bottles per family per year; ^6^ $8/month spent for 14 bottles of Waterguard, school invested $72 per year for waterguard; ^7^ $0.60/per 500 mL bottle lasting 2 months per family; ^8^ 40 2-L bottles @ $0.16 per bottle; ^9^ $0.01/10 L or per sachet treats 10 L, 4 treatments per day; ^10^ $0.05/10 L or per sachet treats 10 L, 4 treatments per day; ^11^ Source says $2.80 for 2; ^12^ Costs for repairs or maintenance paid by households/families since the construction of the latrine; ^13^ Janitorial salary = $15,916, latrine emptying fee = $884; ^14^ Additives include salt, ashes, old batteries, diesel or paraffin to reduce “sludge volume.” Assumed treatments occur 2 times per year; ^15^ ½ roll of toilet tissue per pupil.

**Table 10:** WASH recurring costs in Asian countries.

	Recurring Cost Type	Cost	Unit	Per Person, Per Year 2015 USD	Country	Source
Water Supply	**Operation and maintenance**					
	House connection	$7.30	per person, per year ^1^	$8.31	Unspecified	[60]
Water Treatment and Storage	**Filtration**					
	Ceramic candles for candle filter	$0.90–1.30	per ceramic candle	$0.52–0.76	India	[38]
	Ceramic pot filter	$0.20	median cost per m^3^	$1.83	Unspecified	[60]
	Ceramic pot filter	$7–12	per filter	$2.04–3.49	Cambodia	[38]
	Kanchan ™ arsenic filter (KAF)	$20	per filter, 15 L/h	$0.13 ^2^	Nepal	[25]
	**Water testing**	$60	per month with a replicate	$0.01	India	[49]
Sanitation	**Household sewer connection**	$0.92	per month, per household ^3^	$3.34	India	[48]
	**Operation and maintenance**					
	Bucket latrines	$1.17	per month, per latrine	$0.93	India ^4^	[48]

^1^ Assumes a mean daily water consumption of 100 L per capita for household connection; ^2^ Estimated lifespan of 10 years; ^3^ Centralized system, in urban areas only.; ^4^ Citing: Altaf, 1994 [53].

**Table 11:** WASH recurring costs in Latin American countries.

	Recurring Cost Type	Cost	Unit	Per Person, per Year 2015 USD	Country	Source
Water Supply	**Cost of Water**					
	House connection	$0.08	per 378 L	$0.01	Peru	[66]
	Cost of water in city for those without house connection	$0.38	per 378 L	$0.03	Peru	[66]
	**Operation and Maintenance**					
	House Connection	$10.95	per person, per year ^1^	$12.46	Unspecified	[60]
	House Connection (plus administration)	$16.71	per month	$135	Trinidad	[67]
Water Treatment and Storage	**Disinfection**					
	Chlorine Bottle	$0.09–0.12	per bottle, per family, per month	$0.33–0.44	Haiti	[46]

^1^ Assumes a mean daily water consumption of 100 L per capita for those with house connections.

**Table 12:** WASH recurring costs in unspecified countries.

	Recurring Cost Type	Cost	Unit	Per Person, per Year 2015 USD	Country	Source
Water Supply	**Recurrent costs of water supply hardware**					
	Dug well	$0–4.80	per year ^1^	$6.03	Unspecified ^2^	[56]
	Handpump on drilled well	$0–5.50	per year ^3^	$6.91	Unspecified ^2^	[56]
	House connection	$18.40–57.60	per year ^4^	$23.10–72.32	Unspecified ^2^	[56]
	Rainwater harvesting	$1.70–7.35	per year ^5^	$2.13–9.23	Unspecified ^2^	[56]
	Standpost	$0–6.40	per year ^6^	$8.04	Unspecified ^2^	[56]
	**Operation and maintenance**					
	Private connection	$20,037–40,000	per village, per year ^7^	$15.17–30.64	Unspecified	[48]
	Public standpipe	$15,939.39–26,136.36	per village, per year ^8^	$7.92–12.07	Unspecified	[48]
Water Treatment and Storage	**Filtration**					
	Ceramic filter	$4-5	per replacement ceramicpot element	$0.37–0.46 ^9^	Multiple	[57]
	Ceramic filtration	$3.03	per person, per year	$3.72	Multiple	[33]
	**Disinfection**					
	Chlorination	$0.66	per person, per year	$0.81	Multiple	[33]
	Chlorine bottle	$1	per bottle^10^	$0.15	Multiple	[57]
	Chlorine tablets	$0.001–0.01	per L	$8.01–80.14	Multiple	[57]
	Coagulant-chlorine disinfection system (PuR sachet)	$0.003–0.010	per L	$24.04–80.14	Multiple	[57]
	PuR sachet	$0.25	per sachet	$106.19	Unspecified	[38]
	Flocculation-disinfection	$4.95	per person, per year	$6.07	Unspecified	[33]
	PuR sachet	$0.035	per sachet plus shipping	$34.74	Unspecified	[46]
	Solar disinfection	$0.63	per person, per year	$0.77	Unspecified	[33]
Sanitation	**Operation and Maintenance**					
	Private toilet	$113	per toilet plus septic tank, per year	$6.84	Unspecified	[48]

^1^ Calculated as 0–10% of cost of hardware, annual cost (hardware ranges from $21–48 per person; ^2^ Citing WHO/UNICEF, 2000 [31]; ^3^ Calculated as 0–10% of cost of hardware, annual cost (hardware ranges from $17–55 per person for handpump. ^4^ Calculated as 20%–40% recurrent annual cost of hardware (hardware $92–144 per person; ^5^ Calculated as 5%–15% recurrent annual cost of hardware (hardware ranges from $34–49 per person; ^6^ Calculated as 0–10% recurrent annual cost of hardware (hardware $31–64 per person; ^7^ $20,037 for a small village, 26,890 for a medium village, $40,000 for a large village; ^8^ $15,939.39 for a small village, $1,775,758 for a medium village, $26,136.36 for a large village. ^9^ Assumed 3 year lifespan. ^10^ Can treat >1000 L of water and last months.

**Table 13:** Sources that include successful or theoretical WASH financing models in community or school settings.

**Sources with Successful Financing Models**				
**Financing for WASH in Community or School?**	**Government/Public Financing**	**Private/NGOFinancing**	**User/Household or School Fees**	**Country and Source**
Community	X	X	X	Pakistan [68] *, Ghana [36]
Community	X		X	Zimbabwe [59] ^1^, India [51] ^2^
Community & School	X	X	X	Latin American Countries [30], Ethiopia [26]
School	X	X	X	Kenya [24], Bangladesh [29]
School			X	Kenya [23] ^3^
**Sources with Theoretical Financing Models**				
**Financing for WASH in Community or School?**	**Government/Public Financing**	**Private/NGOFinancing**	**User/Household or School Fees**	**Country and Source**
Community	X		X	Tanzania [41] ^4^
Community & School	X	X	X	Liberia [69]
**Sources with Successful and Theoretical Financing Model**				
**Financing for WASH in Community or School?**	**Government/Public Financing**	**Private/NGOFinancing**	**User/Household or School Fees**	**Country and Source**
Community	X	X	X	Multiple Countries [12,33,39,46,48,50,70,71]
Community	X		X	Unspecified [64] *
Community & School	X	X	X	Multiple [72] *, India [27]
Community	X	X		East and Central Africa [73] *

* Source includes both WASH and relevant non-WASH financing models; ^1^ Community health clubs only, very brief mention of financing; ^2^ Toilets only; ^3^ Handwashing only; ^4^ Pit Emptying only.

**Table 14:** Sources with applicable non-WASH financing models.

Financing Models Included	Government/Public Financing	Private/NGOFinancing	User/Household or School Fees	Country and Source
Poverty Reduction, Health care, Public water utility, Infrastructure, Education, Youth Development, Poverty, Social Safety net, Agriculture	X	X	X	Bangladesh [74]
Water treatment- Arsenic Removal for community water	X		X	India [49]
Healthcare, Insurance	X	X	X	Multiple [75]
Higher Education	X	X	X	Multiple [76]
